# Identification of tau UFMylation *in vitro and in vivo*

**DOI:** 10.1002/alz.092367

**Published:** 2025-01-03

**Authors:** Tingxiang Yan, Benjamin J. Madden, Melissa E. Murray, Dennis W. Dickson, Wolfdieter Springer, Fabienne C Fiesel

**Affiliations:** ^1^ Mayo Clinic, Jacksonville, FL USA; ^2^ Mayo Clinic, Rochester, MN USA

## Abstract

**Background:**

UFMylation is an understudied ubiquitin‐like post‐translational modification (PTM). Like ubiquitin, UFM1 is conjugated to substrates via a catalytic cascade involving a UFM1‐specific E1 (UBA5), E2 (UFC1), and an E3 ligase complex (UFL1, DDRGK1 and CDK5RAP3). UFMylation is reversible, and this is mediated by UFSP2. UFMylation plays essential roles in several biological processes including the DNA damage response, endoplasmic reticulum homeostasis, unfolded protein response, autophagic functions, and immune response. Importantly, UFMylation is also crucial for healthy brain development. Alzheimer’s disease (AD), a complex neurodegenerative disorder, is characterized by the accumulation of pathologic tau and beta‐amyloid proteins and has been associated with abnormalities in the aforementioned processes. Intriguingly, the potential link between AD and UFMylation has not been investigated yet. Considering that pathological tau plays a critical role in AD, and tau also plays an important role for the DNA damage response in neurons under physiological conditions, we speculated that tau itself could be modified with UFM1.

**Method:**

We performed Western blot and Meso Scale Discovery based ELISA to measure the protein level of UFM1, UFSP2 and other UFMylation pathway components in RIPA‐soluble and insoluble fraction in human post‐mortem frontal cortex. To test whether tau can be modified by UFM1, we performed co‐immunoprecipitation (Co‐IP), Proximity Ligation Assays (PLA) and liquid chromatography‐tandem mass spectrometry (LC‐MS/MS).

**Result:**

We found a significant reduction of soluble UFSP2, but an increase of soluble total UFM1 in AD compared to controls. We found that tau interacts with UFL1 and DDRGK1. In addition, using immunoprecipitation of denaturing lysates we found that tau can indeed be covalently modified by UFM1 when overexpressed. We detected strong signal of HA‐UFM1‐tau in neurons but not in negative controls using PLA. Furthermore, we immunoprecipitated denatured UFM1 from human autopsy brain, and detected two specific UFM1‐tau bands with tau antibodies. Together, this data strongly indicates that tau is UFMylated under endogenous conditions. Using LC‐MS/MS, we identified 17 UFMylation sites in tau proteins.

**Conclusion:**

UFMylation is dysregulated in AD; tau protein can be modified by UFM1 both *in vitro* and *in vivo*, which enables a more comprehensive understanding of tau PTMs.

